# Chorea

**Published:** 1905-02-25

**Authors:** 


### CHOREA.

Dr. Archibald Garrod[1] in a recent clinical lecture reviewed the chief features of chorea as seen in its most frequent victims—namely, children before the years of adolescence. He described chorea as a clinical condition of three main elements—motor, paralytic, and cardiac.

The motor element is evidenced by the typical movements, which are essentially ataxic, and differ from the habit-spasms with which they are liable to be confounded in the fact that they are constantly varying, while "tics" appear as a constant repetition of the same muscular contractions. A marked feature of choreic movement is that it is liable to be one-sided, or that if both sides are involved, the affection is less on one side than the other. This hemiplegic distribution, therefore, is to be regarded as evidence of a local cerebral lesion, since, if the manifestations were merely the result of a general toxæmia it might be expected that the degree of involvement would be equal bilaterally.

The paralytic element, though often not marked, is occasionally the first thing to attract attention, and a certain degree of paresis is almost constant in choreic children, evidenced by weakness in one or other leg, or in a diminished strength of grasp. The knee-jerk is sometimes absent, sometimes affected in a way peculiar to this disease; that is to say, it is "hung-up," the limb responds, but instead of the contraction being, as it is in health, momentary, it lasts for an appreciable interval. Dr. Garrod observes that in the course of the disease the knee jerk though previously normal, may subsequently disappear. This is an important practical point in a disease, whose therapeutics so commonly centre round arsenical compounds. The disappearance of this reflex does not necessarily mean that the administration of arsenic, supposing it to have been given, has reached the first stage of peripheral neuritis.

The cardiac element is often organic, but may be only functional. Its mildest form is the irregularity of rhythm often spoken of as "chorea cordis"; it represents nothing more than chorea of the heart's muscle, and is of interest in that it does not, like other choreic movements, disappear during sleep. The author points out very appositely that the heart, unlike the skeletal muscles, is never able to sleep.

The commonest organic lesion of the heart is endocarditis, most often apparent first or only in the mitral valve, and offering the systolic apical murmur of mitral regurgitation. But all the valves may, and the aortic valves only too often are, similarly involved.

A certain degree of myocarditis is almost constant in cases in which the heart is at all affected, and signifies its presence by the signs and symptoms of cardiac dilatation, *i.e.* enlargement of the cardiac dulness in both lateral directions.

Lastly, the pericardium may be attacked, but this is a far less frequent accident than the involvement of the endocardium and heart's muscle. As incidental features of the disease, Dr. Garrod mentions the emotional disturbances which are almost constantly to be noted, either in the direction of laughter or tears. This exaggerated excitability supplies remarkable emotional outbursts on slight provocation, and explains the large part credited, by popular estimation, to fright as a determining factor of the incidence of chorea. The fright, according to this hypothesis, is merely the abnormal response of an already choreic child to a slight stimulus, and the fact that the incriminated alarms are often of the most trifling order, lends colour to this supposition.

As regards the ætiological relationship of chorea and rheumatism Dr. Garrod considers that the former is a manifestation of the latter in a vast majority of cases. Rheumatism in childhood is vitally different from the adult disease in the subordination of the articular features. Chorea is one of a group of symptoms specially prominent in the rheumatism of childhood—a group whose chief members are cardiac lesions, chorea, and the rheumatic nodules found over various bony prominences, such as the elbows, knees, and knuckles, and occasionally in the scalp, tendon-sheaths and elsewhere. The chief importance of these nodules is the indication they afford of the activity of the rheumatic process; they practically never appear without evidence of organic heart disease.

As regards the treatment of chorea, Dr. Garrod inclines to the pessimistic view that chorea is practically insusceptible to any active medication, and advocates the established doctrine of rest. Dr. Clive Riviere, on the other hand, regards with favour the administration of ergot and arsenic. As the result of his experience, he believes that some cases yield to ergot—given as the liquid extract, in doses of 1 to 1½ drachms thrice daily—while others rapidly give way before moderate doses of arsenic—three minims of Fowler's solution thrice daily. Some cases again, recalcitrant to both drugs separately, will improve on a combination of them, while, finally, there is a small minority in which no medication appears to exercise the slightest influence.

[1] Clin. Jour., Feb. 8, 1905.

